# The treatment of melasma with a 785 nm wavelength laser in darker phototypes: An Egyptian experience

**DOI:** 10.1111/srt.13534

**Published:** 2023-12-07

**Authors:** Shady Mahmoud Ibrahim, Beatrice Marina Pennati, Riccardo Stocchi, Tiziano Zingoni

**Affiliations:** ^1^ Dermatology and Venereology, Faculty of Medicine Al‐Azhar University Cairo Egypt; ^2^ El.En. Group Florence Italy

To the Editor,

In women in their 30s/40s with darker phototypes is more common to find melasma. It is an acquired hypermelanotic pathology that appears as irregular, light‐ to dark‐brown‐coloured macules on sun‐exposed skin, especially on the face.^1^, ^2^

The most common treatments are chemical peels, lasers, topical and oral medications.^3^

The goal of laser therapy is to target a particular chromophore that absorbs the laser's wavelength in a specific manner while minimising collateral tissue damage, according to the selective photothermolysis theory.^4^ Specifically, picosecond lasers use pulse lengths much less than the target's thermal relaxation period to achieve a higher peak temperature without inflicting thermal damage to the surrounding tissues. Because of this, the picosecond 785‐nm laser offers a superior safety profile compared to other laser types.^5^

In our experience, 12 Egyptian women were treated for facial melasma. They presented Fitzpatrick phototypes ranging from IV (66.7%) to V (33.3%). They were 27 to 42 years old. A picosecond 785 nm laser handpiece pumped by a QS laser (El.En Group, Florence, Italy) with the possibility of adding a contact sensor was utilised. The spot size ranged from 2 to 6 mm, the fluence from 0.4–2 J/cm^2^, and the frequency from 1 to 5 Hz. Two or three passes were made until the treated lesion turned white. Four laser treatments were performed, separated by 40/45 days, until the skin healed completely from the first treatment. As part of home therapy, a whitening cream with tranexamic acid was also applied. Three months after the last laser treatment, the final evaluation and follow‐up visits took place.

A 5‐point (0‐4) scale—Global Aesthetic International Score, GAIS (from No change/Worsening to Excellent improvement) was implied to evaluate the general treated area improvement. The medical study staff that performed the evaluation assessed that 17% of the patients obtained Excellent improvements, 50% Good improvements, 17% Moderate improvements, 5% Mild improvements and 5% No results. Moreover, at a 3‐month follow‐up (FU) the patients’ comfort/satisfaction was evaluated with a 5‐Point Likert Scale Questionnaire (0 = worse; to 4 = very satisfied). The mean value obtained by this data was 2.4 ± 1.5. A Dermoscopic evaluation (DermLite III Pro; 3Gen, CA, USA) was performed at baseline and at 12‐week FU to detect the pigmentation pattern (unpatterned, reticuloglobular, pseudoreticular/arcuate and honeycomb, and dots/granular) and vascular pattern (presence or absence of telangiectasia) of melasma lesion. Some examples of cases at 3 months FU are reported in Figures 1, 2, 3. The whole study population confirmed a near‐complete resolution of melasma, with no adverse effects after treatment.

**FIGURE 1:**
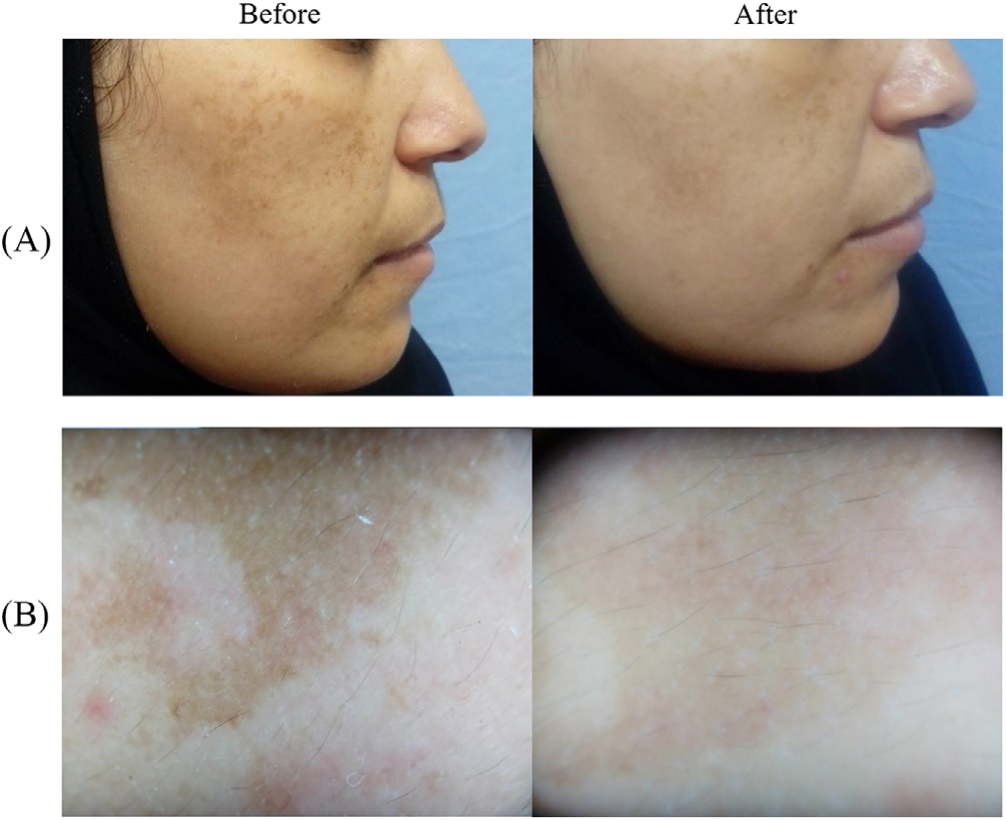
Some of the patients treated for facial melasma with the 785 nm wavelength device are shown. A sensible difference in visible light (A) and with the dermatoscopy (B), between the clinical situation before the treatment and after 3 months of follow‐up, is visible.

**FIGURE 2:**
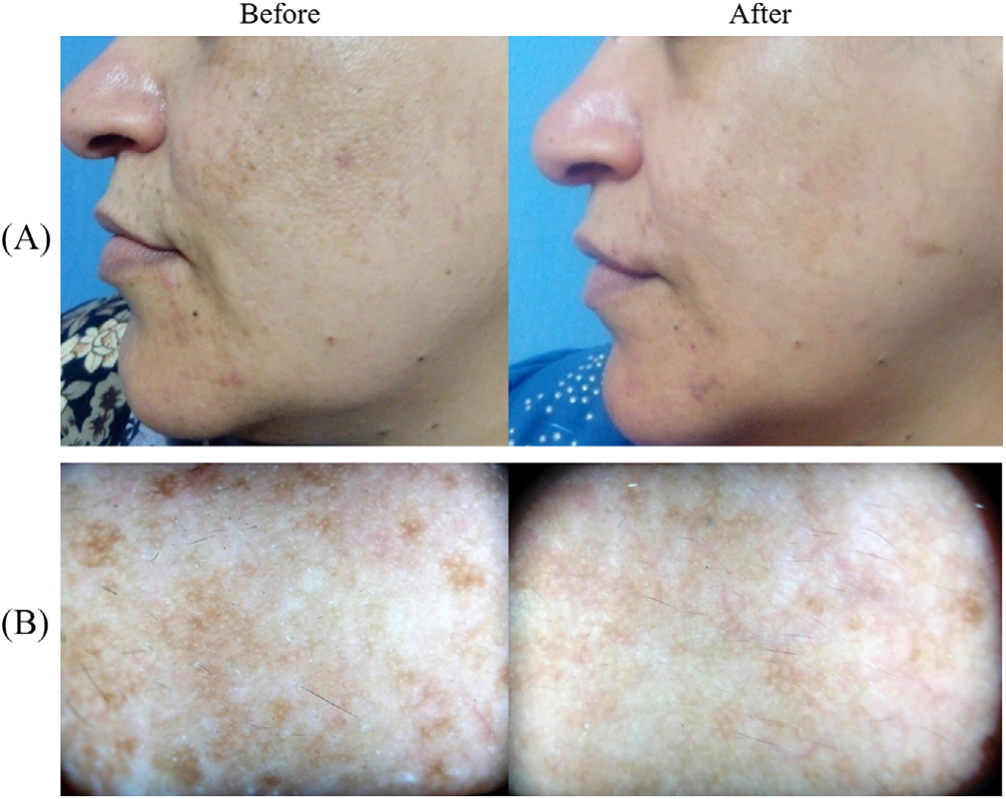
Some of the patients treated for facial melasma with the 785 nm wavelength device are shown. A sensible difference in visible light (A) and with the dermatoscopy (B), between the clinical situation before the treatment and after 3 months of follow‐up, is visible.

**FIGURE 3:**
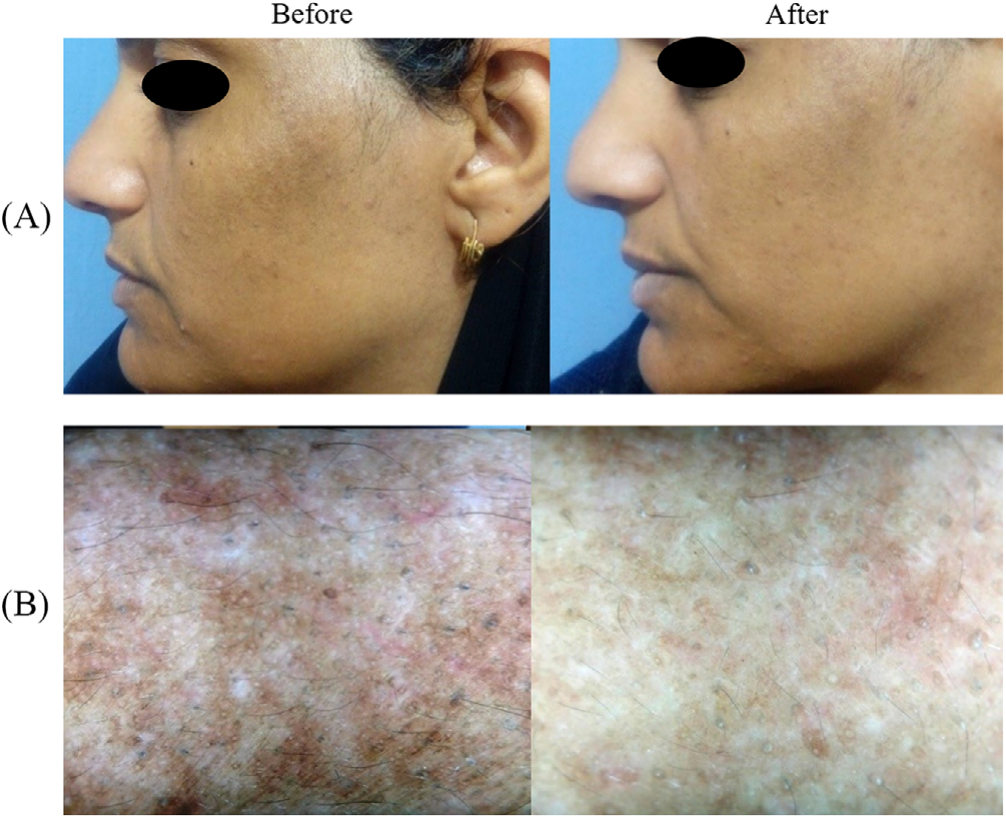
Some of the patients treated for facial melasma with the 785 nm wavelength device are shown. A sensible difference in visible light (A) and with the dermatoscopy (B), between the clinical situation before the treatment and after 3 months of follow‐up, is visible.

In conclusion, we proposed for the first time the application of a 785 nm wavelength emitting device in treating facial melasma pigmentary symptoms in darker phototypes. Indeed, it was used based on its low affinity with the vascular component and so the little possibility of post‐inflammatory effects. This was due to its high affinity for melanin and the characteristic anatomical capillary structure, supporting that this laser could lower the risk of adverse effects and streamline post‐treatment maintenance.

## Data Availability

The data that support the findings of this study are available on request from the corresponding author. The data are not publicly available due to privacy or ethical restrictions.
